# Evaluation of DNA Extraction Methods on Individual Helminth Egg and Larval Stages for Whole-Genome Sequencing

**DOI:** 10.3389/fgene.2019.00826

**Published:** 2019-09-20

**Authors:** Stephen R. Doyle, Geetha Sankaranarayanan, Fiona Allan, Duncan Berger, Pablo D. Jimenez Castro, James Bryant Collins, Thomas Crellen, María A. Duque-Correa, Peter Ellis, Tegegn G. Jaleta, Roz Laing, Kirsty Maitland, Catherine McCarthy, Tchonfienet Moundai, Ben Softley, Elizabeth Thiele, Philippe Tchindebet Ouakou, John Vianney Tushabe, Joanne P. Webster, Adam J. Weiss, James Lok, Eileen Devaney, Ray M. Kaplan, James A. Cotton, Matthew Berriman, Nancy Holroyd

**Affiliations:** ^1^Parasites and Microbes, Wellcome Sanger Institute, Hinxton, United Kingdom; ^2^Department of Life Sciences, Natural History Museum, London, United Kingdom; ^3^Department of Infectious Diseases, College of Veterinary Medicine, University of Georgia, Athens, GA, United States; ^4^Grupo de Parasitologia Veterinaria, Universidad Nacional de Colombia, Bogotá, Colombia; ^5^Nuffield Department of Medicine, University of Oxford, Oxford, United Kingdom; ^6^Department of Pathobiology, School of Veterinary Medicine, University of Pennsylvania, Philadelphia, PA, United States; ^7^Institute of Biodiversity Animal Health and Comparative Medicine, College of Medical, Veterinary and Life Sciences, University of Glasgow, Glasgow, United Kingdom; ^8^Ministry of Public Health, N’Djamena, Chad; ^9^Department of Biology, Vassar College, Poughkeepsie, NY, United States; ^10^Medical Research Council/Uganda Virus Research Institute and London School of Hygiene & Tropical Medicine Uganda Research Unit, Entebbe, Uganda; ^11^Centre for Emerging, Endemic and Exotic Diseases, Department of Pathology and Population Sciences, Royal Veterinary College, University of London, Herts, United Kingdom; ^12^Guinea Worm Eradication Program, The Carter Center, Atlanta, GA, United States

**Keywords:** helminths, genomics, whole-genome sequencing, DNA extraction, low input, diagnostics

## Abstract

Whole-genome sequencing is being rapidly applied to the study of helminth genomes, including *de novo* genome assembly, population genetics, and diagnostic applications. Although late-stage juvenile and adult parasites typically produce sufficient DNA for molecular analyses, these parasitic stages are almost always inaccessible in the live host; immature life stages found in the environment for which samples can be collected non-invasively offer a potential alternative; however, these samples typically yield very low quantities of DNA, can be environmentally resistant, and are susceptible to contamination, often from bacterial or host DNA. Here, we have tested five low-input DNA extraction protocols together with a low-input sequencing library protocol to assess the feasibility of whole-genome sequencing of individual immature helminth samples. These approaches do not use whole-genome amplification, a common but costly approach to increase the yield of low-input samples. We first tested individual parasites from two species spotted onto FTA cards—egg and L1 stages of *Haemonchus contortus* and miracidia of *Schistosoma mansoni*—before further testing on an additional five species—*Ancylostoma caninum*, *Ascaridia dissimilis*, *Dirofilaria immitis*, *Strongyloides stercoralis*, and *Trichuris muris*—with an optimal protocol. A sixth species—*Dracunculus medinensis*—was included for comparison. Whole-genome sequencing followed by analyses to determine the proportion of on- and off-target mapping revealed successful sample preparations for six of the eight species tested with variation both between species and between different life stages from some species described. These results demonstrate the feasibility of whole-genome sequencing of individual parasites, and highlight a new avenue toward generating sensitive, specific, and information-rich data for the diagnosis and surveillance of helminths.

## Introduction

Accurate methods for diagnosis and surveillance of helminth infections are of increasing interest in both human and animal health settings. Such approaches are typically proposed to monitor the presence and ultimately decline of populations targeted by large-scale control measures, such as mass drug administration (MDA) for the prevention and/or treatment of human helminth infections, or prophylactic treatment of domesticated animals. An ideal diagnostic will be sensitive to detect the parasite if in fact present, and specific, to identify the targeted parasite species in the presence of non-target material, such as other parasite species or the host. Ideally, samples taken for diagnostic purposes could be used to gather additional information beyond the presence or absence of a specific parasite, so the same material could be used, for example, to predict how well the infection will respond to drug treatment or how the parasite is related to other endemic or imported parasites. As most parasitic stages of helminths of humans and animals are naturally inaccessible *in vivo* (not accounting for potential availability of some mature stages of helminths following chemo-expulsion, for example, *Ascaris lumbricoides* and *Trichuris trichiura*), a diagnostic should also be informative on non-invasive stages of the parasite, such as eggs deposited in feces, or intermediate stages of the parasite’s life cycle that exist in the external environment.

A key challenge of working with environmental stages of helminth parasites is that they are often immature, for example, eggs or early stage larvae, and extremely small (for example, *Haemonchus contortus* eggs are approximately 75 × 44 μm and *Schistosoma mansoni* miracidia approximately 140 × 55 μm), limiting the amount of accessible material (e.g., DNA) available to be assayed. They are often environmentally resistant, and the same features that naturally protect the DNA from damage prior to reinfection make it difficult to extract DNA. In many cases, they are isolated from host feces and so are susceptible to bacterial contamination or from host tissues and so become contaminated with host DNA. Furthermore, samples may need to be transported efficiently to a laboratory setting without a significant loss of this already limited material. A number of approaches have been tested to preserve macromolecules from individual parasites for transport and storage, including ethanol, RNAlater, and Whatman^®^ FTA^®^ cards, from which robust PCR and microsatellite data could be profiled (Gower et al., 2007; Webster, 2009; Webster et al., 2012; Xiao et al., 2013; Marek et al., 2014; Boué et al., 2017; Campbell et al., 2017). Although, under ideal conditions, the detection of a single DNA molecule is possible, the limited material available per parasite has, to date, largely restricted assaying to a small number of loci, limiting the amount of information obtained from any individual parasite.

Genomic approaches offer an information-rich technology for diagnostic and surveillance applications. Increasing throughput and decreasing costs of whole-genome sequencing has resulted in the recent and steadily growing application of genomics in helminth parasitology, for example, for diagnostic applications, high-throughput amplicon sequencing for helminth species identification and community composition (Avramenko et al., 2015) and the presence of drug resistance alleles (Avramenko et al., 2019) have been described. Although low DNA concentrations are typically prohibitive for genome-wide approaches on individual parasites, a number of studies have successfully used whole-genome amplification on DNA extracted from single larval stages to perform reduced representation (Shortt et al., 2017) and exome (Le Clec’h et al., 2018; Platt et al., 2019) sequencing on miracidia of *Schistosoma* spp., and whole-genome sequencing of *Haemonchus contortus* L3 stage larvae (Doyle et al., 2018) and microfilariae of *Wuchereria bancrofti* (Small et al., 2018). Whole-genome amplification protocols do, however, add considerable expense per sample and can introduce technical artefacts, such as uneven and/or preferential amplification (potentially of contaminant sequences), chimeric sequences, and allele dropout (Tsai et al., 2014; Sabina and Leamon, 2015), that may lead to a reduction in genetic diversity, and in turn, relevance to the original unamplified material. The field of genomics is, however, rapidly advancing toward very low minimum sample input requirements, and single-cell protocols for DNA and RNA sequencing are now available. Such approaches have begun to be used on parasitic species, such as *Plasmodium* spp. (Trevino et al., 2017; Ngara et al., 2018; Reid et al., 2018; Howick et al., 2019), but are yet to be adopted by helminth parasitologists. Although these low-input, high-throughput approaches are not designed—and perhaps not currently suitable—for diagnostic applications, the development of molecular biology techniques for low-input sequencing could aid in the use of genomics for helminth applications. Here, we tested a number of low-input DNA extraction approaches for individual helminth samples stored on Whatman^®^ FTA^®^ cards, followed by low-input library preparation without whole-genome amplification and whole-genome sequencing. A total of five DNA extraction approaches were initially tested, after which the most promising approach was applied to multiple life stages from seven helminth species (with an additional species presented for comparison). The results presented here demonstrate the advancement of low-input whole-genome sequencing and may be broadly applicable to helminth and non-helminth species for which low-input whole-genome sequencing is to be performed. Finally, we discuss our results in the context of helminth diagnostics and surveillance.

## Methods

### Sample Collection

Samples representing accessible, immature life stages of a total of eight helminth species were tested, the collection of which is described below. A number of protocols and substrates or solutions have been described for the safe, effective storage of samples for downstream molecular biology applications; we chose Whatman^®^ FTA^®^ cards as a substrate to store and transport parasite material, as they are a relatively cost-effective method for storing samples without the need for specialized storage and transport conditions, for example, a cold chain from collection to sequencing. Removal of the logistical hurdles and their associated costs is particularly important for collection of specimens from endemic regions, for example, *S. mansoni* miracidia that were isolated from patients and purified in Africa before transported and stored on FTA cards for down-stream processing the UK as described here.

*Ancylostoma caninum*: Fresh feces from a research purpose-bred laboratory beagle (University of Georgia, AUP A2017 10-016-Y1-A0) infected with the Barrow isolate (drug-susceptible isolate from Barrow County Georgia, USA) were collected and made into a slurry with water, filtered through 425- and 180-µm sieves, and centrifuged at 2500 rpm for 5 min, after which the supernatant was discarded. Kaolin (Sigma-Aldrich, St. Louis, MO) was then added and resuspended in sodium nitrate (SPG 1.25–1.3) (Feca-Med^®^; Vedco, Inc. St Joseph, MO, USA). The tube was then centrifuged at 2500 rpm for 5 min, after which the supernatant was passed through a 30-µm sieve and rinsed with distilled water, and reduced to a volume of 10 to 15 mL. The volume was adjusted to one egg per 5 µl using distilled water. The eggs were stored at room temperature for 2 h before placing them onto the Whatman^®^ FTA^®^ cards. Eggs were also placed onto Nematode Growth Medium (NGM) plates (Sulston and Hodgkin, 1988) and incubated at 26°C to obtain the first-stage (L1) larvae. After 48 h, larvae were rinsed off the plate with distilled water and centrifuged at 1000 rpm for 5 min. Larvae were counted, and the concentration adjusted to one larva per 5 µl. The larvae were stored at room temperature for 2 h before placing them onto the Whatman^®^ FTA^®^ cards. To obtain third-stage (L3) larvae, eggs were isolated from fresh feces from a research purpose-bred laboratory beagle (University of Georgia, AUP A2017 10-016-Y1-A0) infected with the Worthy isolate (Worthy 3.1F3Pyr; multiple-drug resistant isolate originally isolated from a greyhound dog, Florida, USA). Eggs were placed onto NGM plates and incubated at 26°C. After 7 days, larvae were rinsed off the plate with distilled water and centrifuged at 1000 rpm for 5 min. Larvae were counted, and the concentration adjusted to one larva per 5 µl. The larvae were stored at room temperature for 2 h before placing them onto the Whatman^®^ FTA^®^ cards.

*Ascaridia dissimilis*: Eggs of *A. dissimilis* (Isolate Wi: North Carolina, USA) were isolated from excreta of experimentally infected turkeys. Water was added to the excreta and made into a slurry, which was filtered using a 425-µm and 180-µm sieve to remove large debris. The remaining particulates were placed into 50-mL centrifuge tubes and centrifuged at 433*g* for 7 min. Supernatant was removed, and the pellet was resuspended in a saturated sucrose solution with a specific gravity of 1.15. The suspension was centrifuged as before, and eggs were isolated from the top layer. Eggs were rinsed over a 20-µm sieve with water to remove residual sucrose and then concentrated to one egg per 5 µl using deionized water. Multiple 5-µl aliquots of the egg solution were dispensed using a micropipette onto the Whatman^®^ FTA^®^ card.

*Dirofilaria immitis*: Blood was taken from a dog infected with the macrocyclic lactone (ML)-resistant Yazoo strain (Yazoo: originally isolated from a dog in Yazoo City, Mississippi, USA; see Maclean et al. (2017) for complete history). To obtain microfilariae, blood was collected in heparin tubes and centrifuged for 30 min at 2500 rpm, after which the supernatant was discarded. The pellet was suspended in 3.8% sodium citrate (Sigma-Aldrich, St. Louis, MO) and 15% saponin (Sigma-Aldrich) was added in a 1:7 dilution. The tube was then vortexed and centrifuged for 30 min at 2500 rpm, after which the supernatant was discarded and the pellet resuspended in 3.8% sodium citrate to the original blood volume, vortexed, and then centrifuged for 4 min at 2500 rpm. The pellet was then resuspended and mixed in a 1:9 solution of 10𝖷 phosphate-buffered saline (PBS) (Thermo Fisher Scientific, Waltham, MA) and distilled water. The tube was then centrifuged for 4 min at 2500 rpm, and the pellet resuspended in PBS. The microfilariae were then counted and adjusted accordingly to have one microfilaria per 5 µl and stored at room temperature for 2 h before placing them onto the Whatman^®^ FTA^®^ cards.

*Dracunculus medinensis*: Individual L1 samples were obtained as progeny of an adult female worm manually extracted from an infected dog in Tarangara village, Chad (9.068611 N, 18.708611 E) in 2016. This extraction forms part of the standard containment and treatment procedure for Guinea worm infections, as agreed upon and sanctioned by the World Health Organization and country ministries of health. The adult worm was submerged in ethanol in a microcentrifuge tube for storage; L1 stage progeny that were found settled on the bottom of the tube were collected for analysis.

*Haemonchus contortus*: Eggs representing the F5 generation of a genetic cross (described in Doyle et al. (2018)) were collected from fresh feces from experimentally infected sheep housed at the Moredun Research Institute, UK. All experimental procedures were examined and approved by the Moredun Research Institute Experiments and Ethics Committee and were conducted under approved UK Home Office licenses (PPL 60/03899) in accordance with the Animals (Scientific Procedures) Act of 1986. Briefly, feces were mixed with tap water and passed through a 210-µm sieve, then centrifuged at 2500 rpm for 5 min in polyallomer tubes. The supernatant was discarded, before adding kaolin to the fecal pellet, vortexing, and resuspending in a saturated salt solution. After centrifugation at 1000 rpm for 10 min, the polyallomer tube was clamped to isolate eggs, which were collected on a 38-µm sieve and rinsed thoroughly with tap water. Eggs were incubated on NGM plates at 20^o^C for 48 h to hatch to L1 stage larvae. In addition to freshly collected material, eggs collected in the same manner then stored at −20^o^C, from a previous generation of the cross, were also tested. Eggs and L1 larvae were resuspended in PBS and spotted onto Whatman FTA cards in 3 µl per egg/L1.

*Schistosoma mansoni*: Three collections of *S. mansoni* samples were used in this work. The first were field samples collected from humans on Lake Victoria fishing villages in Uganda as part of the LaVIISWA trial (Sanya et al., 2018). Ethical approval for this trail was given by the Uganda Virus Research Institute (reference number GC127), the Uganda National Council for Science and Technology (reference number HS 1183), and the London School of Hygiene & Tropical Medicine (reference number 6187). Parasite eggs were collected from participants’ stool samples using a Pitchford-Visser funnel, washed with mineral water until clean, and transferred into a petri dish with water to be hatched in direct sunlight. After hatching, the miracidia were picked in 2-µl water using a pipette and placed on a Whatman^®^ FTA^®^ card for storage. The second were field samples collected as part of a repeated cross-sectional study of MDA exposure in school children in Uganda. Patient enrolment, including written consent, and sample collection have been described previously (Crellen et al., 2016). Ethical approvals for this study were granted by the Uganda National Council of Science and Technology (MoU sections 1.4, 1.5, 1.6) and the Imperial College Research Ethics Committee (EC NO: 03.36. R&D No: 03/SB/033E). Host stool was sampled 1 to 3 days prior to treatment with praziquantel (40 mg/kg) and albendazole (400 mg). A Pitchford-Visser funnel was used to wash and filter stool to retain parasite eggs. The filtrate was kept overnight in water and hatched the following morning in sunlight. Individual miracidia were isolated with a 20-μL pipette and transferred into petri dishes of nuclease-free water twice before spotting onto Whatman^®^ FTA^®^ cards. The third source of miracidia was derived from the livers of experimentally infected mice kept to maintain the *S. mansoni* life cycle at Wellcome Sanger Institute. Mouse infection protocols were approved by the Animal Welfare and Ethical Review Body (AWERB) of the Wellcome Sanger Institute, and in accordance with the UK Home Office approved project license P77E8A062. The AWERB is constituted as required by the UK Animals (Scientific Procedures) Act 1986 Amendment Regulations 2012. Balb/C mice (6–8 weeks old) were infected with 250 cercariae, after which livers were collected on day 40 postinfection. Eggs were isolated from the liver tissues using collagenase digestion followed by percoll gradient, and were washed well with sterile PBS, before being hatched in sterile conditioned water. The hatched individual miracidia were spotted onto Whatman^®^ FTA^®^ cards.

*Strongyloides stercoralis*: The *S. stercoralis* UPD strain and the isofemale isolate PVO1 were maintained in purpose-bred, prednisone-treated mix breed dogs according to protocol 804883 approved by the University of Pennsylvania Institutional Animal Care and Use Committee (IACUC), USA. IACUC-approved research protocols and all routine husbandry care of the animals were conducted in strict accordance with the Guide for the Care and Use of Laboratory Animals of the National Institutes of Health, USA. Feces were collected, moisturized, and mixed with equal volume of charcoal and cultured at 22^o^C in 10-cm plates (Lok, 2007). Post-parasitic stage one and two larvae (L1/L2), free-living male and female adults, and infective third-stage (L3) were isolated by the Baermann technique after 24 and 48 h, and 6 days in these charcoal coprocultures, respectively (Lok, 2007; Jaleta et al., 2017). For this study, free living females (FL), L1 and L3 larvae were collected from the Baermann funnel sediments and washed three times using PBS.

*Trichuris muris*: Infection and maintenance of *T. muris* was conducted as described (Wakelin, 1967). The care and use of mice were in accordance with the UK Home Office regulations (UK Animals Scientific Procedures Act 1986) under the Project license P77E8A062 and were approved by the institutional AWERB. Female SCID mice (6–10 wk old) were orally infected under anaesthesia with isoflurane with a high dose (n = 400) of embryonated eggs from *T. muris* E-isolate. Mice were monitored daily for general condition and weight loss. At day 35 postinfection, mice were killed by exsanguination under terminal anesthesia, after which adult worms were harvested from cecums. Adult worms were cultured in RPMI 1640 supplemented with 10% fetal calf serum (v/v), 2 mM L-glutamine, penicillin (100 U/mL), and streptomycin (100 mg/mL; all Invitrogen), for 4 h or overnight, and eggs were collected. The eggs were allowed to embryonate for at least 6 weeks in distilled water, and infectivity was established by worm burden in SCID mice. *T. muris* eggs were hatched to produce sterile L1 larvae using 32% sodium hypochlorite in sterile water for 2 h at 37°C with 5% CO_2_. Eggs were washed with RPMI 1640 supplemented with 10% fetal calf serum (v/v), 2 mM l-glutamine, penicillin (100 U/mL), and streptomycin (100 mg/mL; all Invitrogen), and incubated at 37°C with 5% CO_2_ for 4 to 5 days until they hatched.

For each species, unless otherwise stated, pools of individuals were washed in sterile PBS, before being transferred to a petri dish. Individuals were identified under the microscope, after which 5-µl PBS containing an individual parasite was transferred onto a Whatman^®^ FTA^®^ card and dried for a minimum of 20 min at room temperature prior to storage or shipping to the Wellcome Sanger Institute, UK. The Whatman^®^ FTA^®^ cards with samples spotted were stored in a clean plastic bag in the dark at room temperature prior to analysis.

### DNA Extraction

Five DNA extraction methods were tested for their ability to isolate and purify DNA compatible with whole-genome sequencing approaches. The choice of approaches was not systematic or comprehensive, but was available to us at the Wellcome Sanger Institute as low-input DNA extraction approaches that had the potential for high-throughput and/or automatic extraction protocols to generate sequencing libraries suitable for whole-genome sequencing. Despite the arbitrary choice of extraction kits, the comparison of multiple kits with multiple helminth species provides a unique data set that is not easily achieved at this scale outside of a research and development environment, and forms the basis for further comparison with other low-input kits as they become available.

To extract DNA, the sample spots on FTA cards were punched out manually into 96-well plates using either a Harris Punch or autonomously using robotics. The DNA extraction was carried out for each method as described below:

1. *Nexttec* (NXT): Extraction using the Nexttec 1-step DNA Isolation Kit for Tissues & Cells (cat: 10N.904; Waendel Technology Limited, UK) was performed according to manufacturer’s guidelines. Proteinase lysis buffer (75 µl) was used for digestion.
2. *Bloodspot* (BSP): The bloodspot extraction was derived from the QIAamp DNA Investigator Kit (cat: 51104/51106; Qiagen), following the “DNA Purification from Dried Blood Spots (QIAamp DNA Mini Kit)” protocol according to manufacturer’s guidelines.
3. *CGP* (CGP): Extraction using the CGP protocol (Moore et al., 2018) involved adding 30 µl of lysis buffer (1.25 µg/mL of Protease reagent (Qiagen; cat 19155) in Tris HCl pH 8.0, 0.5% Tween 20, 0.5% NP40) to individual Whatman^®^ FTA^®^ punches in a 96-well PCR plate. The samples were incubated at 50°C for an hour followed by protease inactivation by heating the samples to 75°C for 30 min.
4. *ForensicGem* (FGM): Extraction using the ForensicGEM Universal DNA extraction kit (cat: FUN0100; ZyGem) was performed according to the manufacturer’s guidelines.
5. *PicoPure* (PIP): Extraction using the ARCTURUS^®^ PicoPure^®^ DNA Extraction Kit (cat: KIT0103; ThermoFisher) was performed according to the manufacturer’s guidelines, using 75 µl of the extraction buffer.

When the extracted DNA from all the above methods was present in a volume greater than 25 µl, samples were cleaned with Agencourt AMPure XP beads (Beckman-Coulter) and eluted in 25-µl nuclease-free H_2_O. The entire DNA samples were used downstream to make sequencing libraries. A summary of the number of species, life stages, and conditions tested, is presented in **Table 1**. The differences in the number of samples per species and per assay were largely dependent on the samples available to us, sampled across different times, and sometimes, sampled for different purposes, for example, while most test conditions contained between 6 and 10 replicates, the large number of *D. medinensis* samples (n = 129 for one condition) were prepared specifically for a different study but we have included it for comparative purposes, whereas the *S. mansoni* samples (n = 168 for five conditions) were readily available in-house at the Wellcome Sanger Institute, and thus were first used for further validation before samples from other species that required collection from live animal hosts.

**Table 1:** Summary of the species, life stages, and conditions tested using one or more of the DNA extraction approaches followed by whole-genome sequencing.

				Samples sequenced per DNA extraction approach				
Species	Species ID	Life stage	Life stage ID	BSP	CGP	FGM	NXT	PIP
*Haemonchus contortus*	HC	Egg	EGG	10	10	4	10	10
	HC	Egg (frozen)	EGGf	6	6	0	6	6
	HC	Larval stage L1	LS1	10	10	4	10	10
*Schistosoma mansoni*	SM	Miracidia	MIR	59	36	10	51	12
*Ancylostoma caninum*	AC	Egg	EGG	0	8	0	0	0
	AC	Larval stage L1	LS1	0	8	0	0	0
	AC	Larval stage L3	LS3	0	8	0	0	0
*Ascaridia dissimilis*	AD	Egg	EGG	0	8	0	0	0
*Dirofilaria immitis*	DI	Microfilaria	MFL	0	8	0	0	0
*Strongyloides stercoralis*	SS	Free living females	FL	0	8	0	0	0
	SS	Larval stage L1	LS1	0	8	0	0	0
	SS	Larval stage L3	LS3	0	8	0	0	0
*Trichuris muris*	TM	Egg	EGG	0	8	0	0	0
	TM	Egg (bleached)	EGGb	0	8	0	0	0
	TM	Larval stage L1	LS1	0	8	0	0	0
*Dracunculus medinensis*	DM	Larval stage L1	LS1	0	0	0	129	0
Total samples per kit (n = 497 total)				85	150	18	206	38

### Library Preparation and Sequencing

DNA sequencing libraries for all samples were prepared using a protocol designed for library preparation of laser capture microdissected biopsy (LCMB) samples using the Ultra II FS enzyme (New England Biolabs) for DNA fragmentation as previously described (Lee-Six et al., 2019). A total of 12 cycles of PCR were used (unless otherwise stated in **Table S1**) to amplify libraries and to add a unique 8-base index sequence for sample multiplexing. Prior to sequencing, library concentration was determined using a fluorometric dsDNA quantification assay (AccuClear^®^ Ultra High Sensitivity dsDNA Quantitation Kit; Biotium) following the manufacturer’s instructions, and measured using a FLUOstar Omega fluorescence plate reader (BMG Labtech). These data were used to normalized the concentration of library DNA for multiplexing.

Multiplexed libraries were sequenced using the Illumina MiSeq platform with V2 chemistry 150 bp paired end (PE) reads. The *D. medinensis* samples were sequenced as part of a different study using the HiSeq 2500 platform with V4 chemistry 125 bp PE reads. Metadata for each sample, including sample IDs, sequencing lane IDs, ENA sample accession numbers, and data generated are described in **Table S1**. Raw sequence data used in this study is available under the European Nucleotide Archive (ENA; https://www.ebi.ac.uk/en) study ID ERP114942.

### Analysis

We performed sufficient low coverage sequencing on each sample to enable us to identify: (i) the proportion of on-target mapped reads, (ii) the proportion of duplicate reads, i.e. library artefacts, and (iii) the proportion of off-target contaminant reads. On-target reads are defined as reads that mapped to the genome of the species from which DNA extraction was performed, and thus, is a proxy for the specificity of the experiment. Similarly off-target reads are defined as sequencing reads that do not map to the genome of the species from which DNA was extracted, but are derived from putative contaminants. The presence and proportion of contaminant-derived sequencing reads in each sequencing library was further analyzed using the kmer-based classification tool, kraken (v0.10.6-a2d113dc8f) (Wood and Salzberg, 2014), which we have used to assign taxonomic labels to raw sequencing data. Kmers from raw reads are compared with a kraken database (custom database built at WSI containing human [GRCh38] and mouse [GRCm38] genomes, all plasmid, bacterial, and viral genomes, as well as all Illumina adapters, from NCBI at the date of generation [v.pi_qc_2015521]); any raw sequencing reads that match kmers present in the database are defined as contaminants, whereas reads that do not match the database are defined as “kraken_unassigned.” As no helminth genomes are present in the kraken database, these reads of interest will be in the kraken_unassigned category, for example, a high proportion of “kraken_unassigned” reads are indicative of low contamination (or at least low known contaminants present in the kraken database).

Reference genomes from each of the test species were obtained from the helminth genome repository WormBase Parasite (Howe et al., 2017) Release 12. Raw sequence data for each species were mapped to their respective reference genome (which included the mitochondrial genome) using *bwa* (v0.7.17-r1188) (Li, 2013) *mem* using default parameters (with the inclusion of -Y and -M options to use soft clipping for supplementary alignments, and to mark shorter hits as secondary, respectively), after which duplicate reads were marked using *Picard* (v2.5.0; https://github.com/broadinstitute/picard). *Samtools* (v1.3) flagstat and *bamtools* (v 2.3.0) stats were used to characterise the outcome of the mapping, the results of which were collated using *MultiQC* v1.3 (Ewels et al., 2016). Data were manipulated and visualized in the *R* (v3.5.0) environment using the following packages: *ggplot2* (https://ggplot2.tidyverse.org/), *patchwork* (https://github.com/thomasp85/patchwork), and *dplyr* (https://dplyr.tidyverse.org/).

The relative ratio of the mitochondrial to nuclear genome was calculated using *bedtools* (v2.17.0) (Quinlan and Hall, 2010) *makewindows* and *samtools* (v1.6) *bedcov*. This was performed for each life stage of each species described in **Table 2**, whereby average read coverage from the mapped reads (bam files) were determined for the autosomes (characterised as defined chromosomes for *S. mansoni* and *H. contortus*, otherwise, the longest 10 scaffolds for the remaining species were used) and mitochondrial genomes. These data were used to compare the theoretical multiplexing that could be performed per species, either for whole-genome sequencing to achieve 30× genome-wide coverage, or alternatively, sufficient sequencing to achieve 100× coverage of the mitochondrial genome (see **Table 2** for results). Here, these data were calculated based on Illumina high-throughput sequencing using the NovaSeq 6000 system with S4 2 × 150 bp PE chemistry generating 2.5 terabases (Tb) of data and accounting for 85% mapping rate. However, to aid in the design of experiments for other species and sequencing platforms, the number of samples that can be multiplexed can be determined by:

Multiplex (WGS) =Sequencer lane capacity (bp)[ genome size (bp) ] × [ target genome coverage ] × [ 100average mapping efficiency (%)]

**Table 2:** Breakdown of sequencing strategies per species based on whole-genome sequencing at 30× coverage and whole-genome sequencing to achieve 100× whole mitochondrial genome coverage.

Species / stage	Genome assembly size ^1^	Number of samples that can be multiplexed ^2^	mtDNA / nuclear ratio ^3^	Sampled multiplexed targeting mtDNA at 100× coverage ^2^	Nuclear genome coverage per 100× mtDNA genome
*Schistosoma mansoni* *miracidia*	409	173	142.77	7418	0.70
*Haemonchus contortus egg*	283	250	70.76	5313	1.41
*Haemonchus contortus* L1	283	250	44.55	3345	2.24
*Dracunculus medinensis* L1	103	688	70.81	14608	1.41
*Dirofilaria immitis* microfilaria	88	805	14.27	3446	7.01
*Strongyloides stercoralis* FL	42	1687	11.06	5597	9.04

^1^Genome size: Obtained from Wormbase Parasite v12.

^2^Multiplex: total number of samples per NovaSeq 6000 sequencing run with S4 2 *×* 150 bp PE chemistry generating 2.5 Tb of data and accounting for 85% mapping rate. Theoretically possible, but may be limited by barcoding available.

^3^mtDNA/nuclear ratio: based on coverage of mitochondrial and nuclear-derived sequencing reads, normalized to the nuclear genome coverage.

Where the “genome size” is the estimated number of base pairs in the genome, the “targeted genome coverage” is the intended number of reads covering every base pair of the genome, and the “average mapping efficiency” is the typical proportion of mapped reads from a sequencing library. This latter metric is difficult to predict before performing any sequencing, however, knowledge of this from prior sequencing experiments can improve the actual coverage achieved by applying this scaling factor.

This equation can be extended to low-coverage whole-genome sequencing to target a defined mitochondrial DNA coverage using the following:

Multiplex (mtDNA)=Sequencer lane capacity (bp)[ genome size (bp) ] × [ 1mtDNA to nuclear ratio ] × [ 100average mapping efficiency (%)] × [ target mtDNA coverage ]

where “mtDNA to nuclear ratio” is the number of mitochondrial genomes per nuclear genome, which can be determined by relative reads counts performed here, or by alternative molecular approaches, such as qPCR.

The code to reproduce the analysis and figures for this manuscript is described in https://github.com/stephenrdoyle/helminth_extraction_wgs_test.

## Results and Discussion

**Figure 1:**
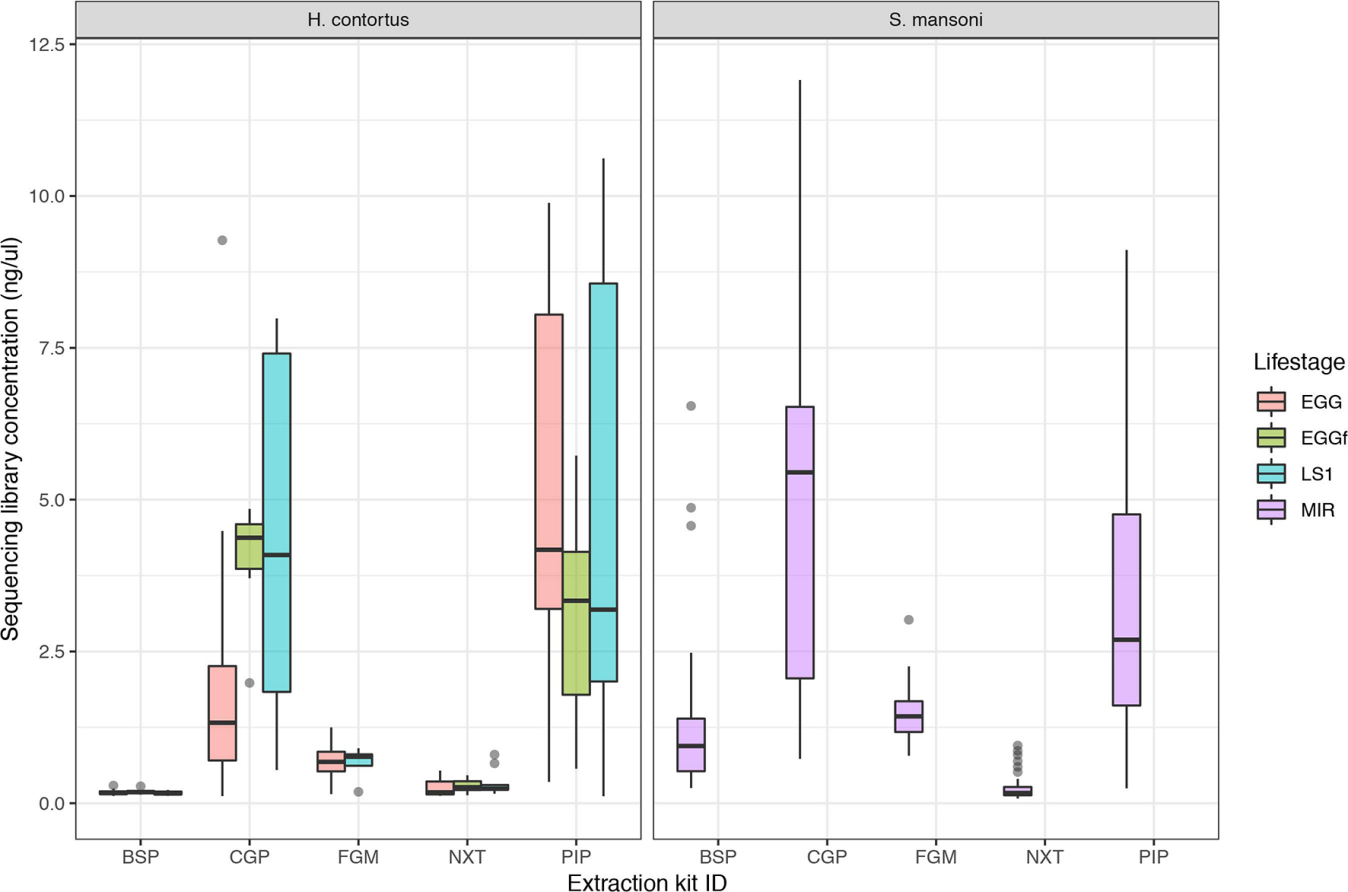
Comparison of sequencing library concentration (ng/µl) as a proxy for relative DNA extraction efficiency for the five DNA extraction protocols tested. DNA from multiple life stages were extracted, including from freshly collected egg (EGG), frozen eggs (EGGf), and L1 stage larvae (LS1) of *Haemonchus contortus*, and miracidia (MIR) of *Schistosoma mansoni*. The number of samples in each comparison is presented in **Table 1**, and the raw data used are described in **Table S1**.

The aim of this work was to determine the feasibility and efficiency of using a low input DNA extraction and library preparation approach for whole-genome sequencing of individual egg and larval stages of helminth parasites. We have targeted immature life stages that are found in the environment for which it is possible to collect samples non-invasively. We first tested our approaches on the nematode *H. contortus* and the trematode *S. mansoni*. Five approaches were tested using single egg (fresh and frozen) and L1 of *H. contortus*, and miracidia of *S. mansoni*. Attempts to quantify the raw DNA extractions were inconsistent and largely unsuccessful, due to the extremely low DNA yield per extraction; in the life stages tested, we expect this yield to be in the range of 10 to 100 s of pg of DNA per sample. Quantification of the sequencing libraries of these extractions did however yield sufficient DNA, and revealed significantly more DNA recovered using the CGP and PIP extractions than the other three protocols (**Figure 1**). Although not a direct measure, sequencing library concentration is likely a sufficient proxy for DNA extraction efficiency, given that the library preparation was standardized across all samples.

We determined the success of the library preparation protocols by comparing (i) the proportion of reads mapped to the genome, representing “on-target” mapping as a measure of specificity (**Table S1**: mapped_reads_percent); (ii) the proportion of reads that matched a “contamination” database, which did not contain helminth genome sequences, and thus represented DNA derived from “off-target” sources such as host or bacterial species commonly present in these samples; those reads that do not match the contamination database are defined as “kraken_unassigned” and are putative helminth-derived reads (**Table S1**: kraken_unassigned_percent); and (iii) the proportion of duplicate reads, which typically represent library preparation artefacts due to over-amplification of DNA during PCR (**Table S1**: duplicate_reads_percent).

**Figure 2:**
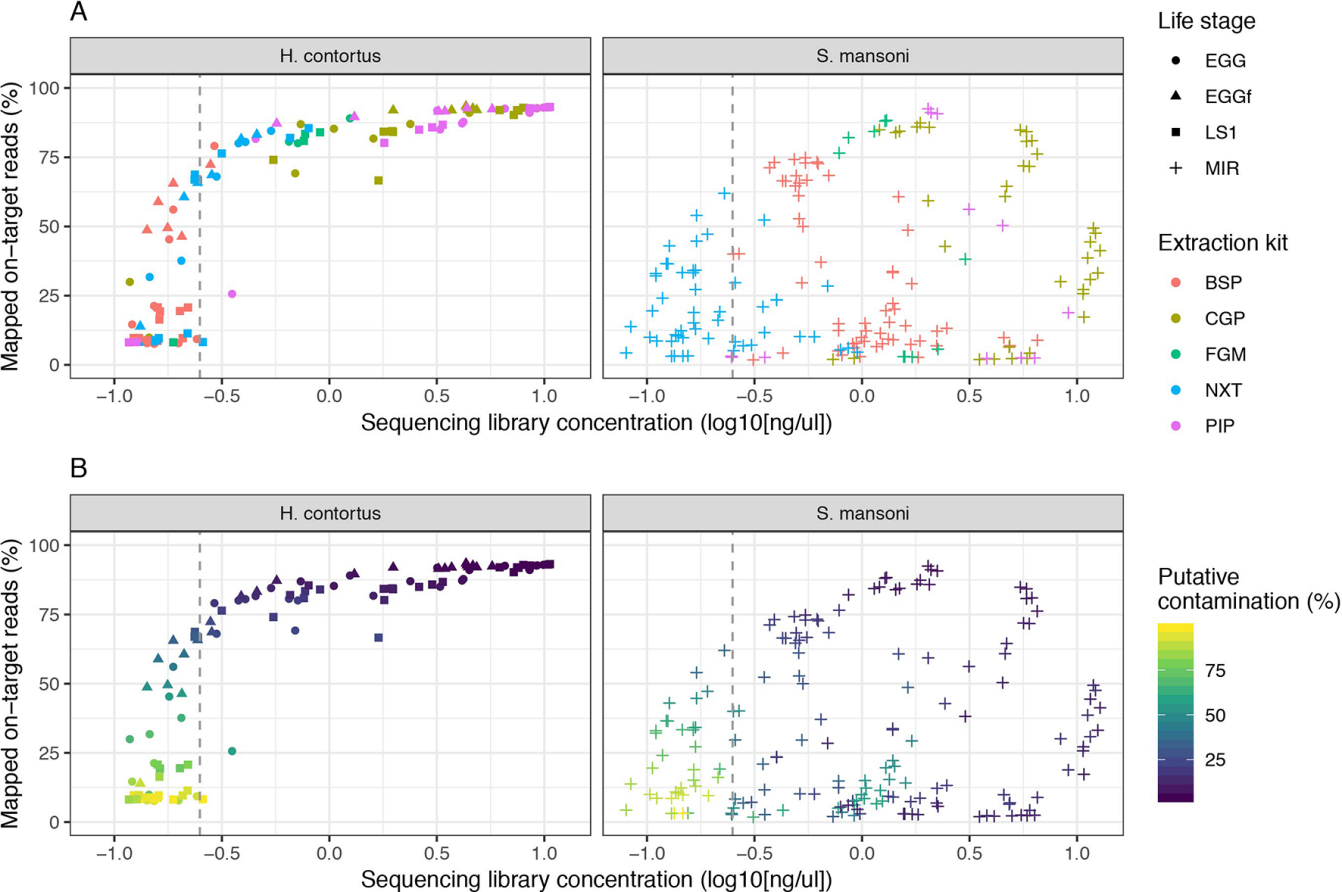
Impact of sequencing library concentration on mapped on-target reads for five low-input DNA extraction protocols. Each point represents an individual sequencing library sample, colored by extraction protocol **(A)** or proportion of putative contaminating reads (100 - percent_unclassified_reads) **(B)**. The dashed grey vertical line represents a library concentration of 0.25 ng/µl. Extraction protocols were performed on *Haemonchus contortus* eggs (EGG), frozen eggs (EGGf), and L1 (LS1) stages, and *Schistosoma mansoni* miracidia (MIR). The number of samples in each comparison is presented in **Table 1**, and the raw data used are described in **Table S1**.

We examined the impact of library strength on mapping frequency for all DNA extraction protocols tested (**Figure 2**). For *H. contortus*, a clear correlation between these two variables were observed, with an inflection point at approximately 0.25 ng/µl, below which the proportion of reads mapped rapidly decreased toward 0 (**Figure 2A**). Similarly, below this point, the proportion of reads classified as contamination increased in frequency (**Figure 2B**). There was, however, a distinct difference between *H. contortus* and *S. mansoni* in the overall proportion of reads mapped, and the frequency of contaminating reads, with greater variation in both parameters in the *S. mansoni* samples. While some of this variation may reflect differences in extracting DNA from the two species, the majority of *S. mansoni* MIR samples were isolated and aliquoted onto FTA cards under less clean conditions in the field and stored for between 2 and 5 years before processing, as compared with the laboratory prepared *H. contortus* samples that were collected and prepared directly from a current infection (EGG and LS1) or within the previous 6 months (EGGf) of processing. We achieved some on-target mapping for all extraction kits tested (**Figure S1A**); however, significant variation was observed between approaches. BSP and NXT generally performed poorly, with either low (median = 19.42; median absolute deviation [MAD] = 16.50) or significant variance (median = 66.20; MAD = 26.18) in mapping frequency between samples observed, respectively. PIP performed consistently well with high mapping rates across all stages in *H. contortus* (median = 90.30; MAD = 3.96); however, it was poor in *S. mansoni* (median = 11.00; MAD = 12.73). The duplication rate of all conditions were within an acceptable low range (median = 0.46, MAD = 0.4), with only 2% of samples having greater than 5% duplicate reads (**Figure S1B**). This suggests that the low DNA input did not noticeably impact duplication rates, and therefore, with greater sequencing depth of the same sequencing libraries, we would expect unbiased genome-wide coverage (at least relative to sequencing of libraries derived from higher DNA input). CGP and FGM performed most consistently between stages and species; however, FGM had higher variance and duplication rates relative to CGP across all samples tested. Considering the higher library strength (suggestive of greater DNA recovery efficiency), consistent mapping efficiency, and the cost effective protocol, we chose the CGP extraction to explore further.

**Figure 3:**
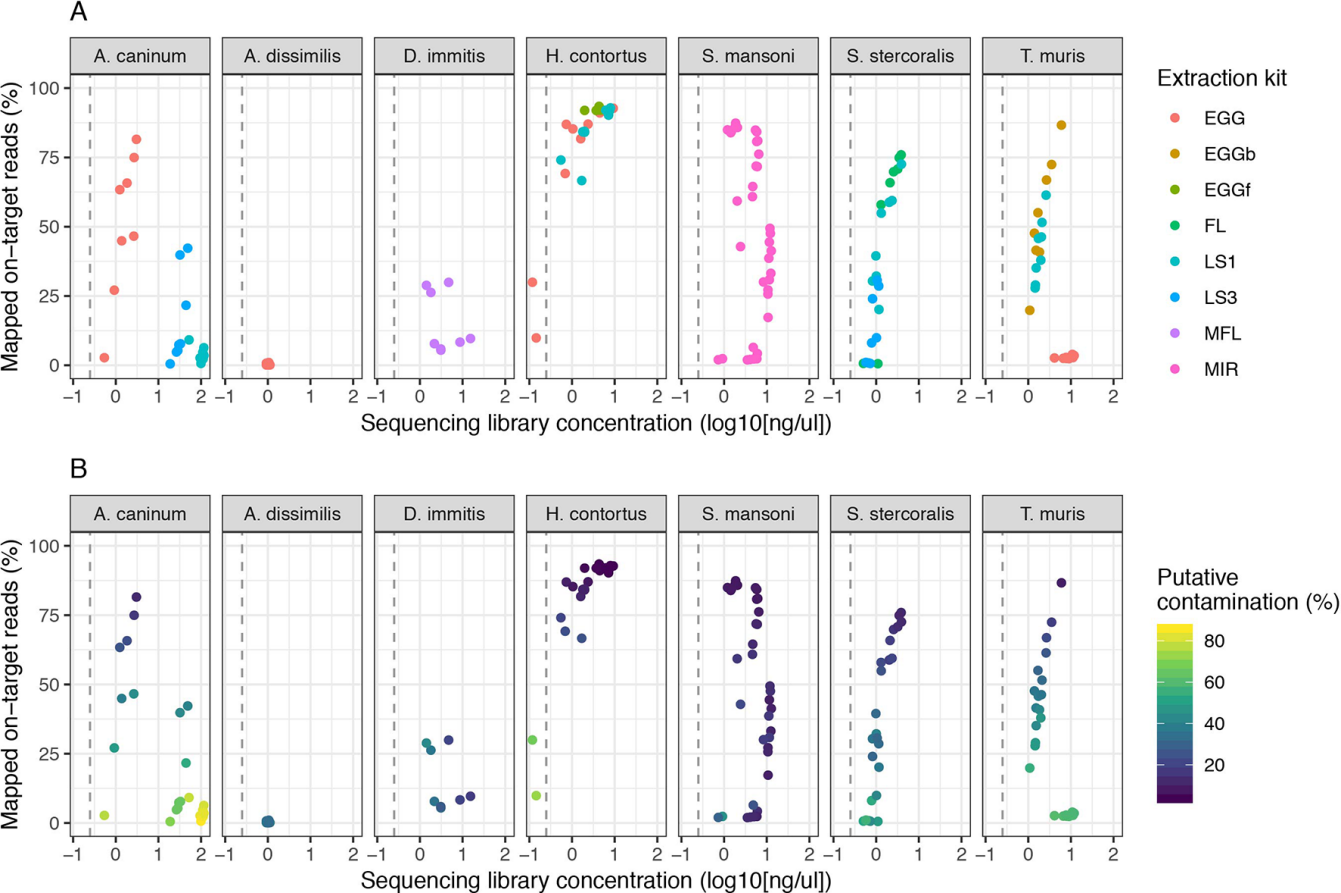
Impact of sequencing library concentration on mapped on-target reads from multiple life stages of seven helminth species. Each point represents an individual sequencing library samples, colored by life stage **(A)** or proportion of putative contaminating reads (100 - percent_unclassified_reads) **(B)**. DNA was extracted from all samples using the CGP protocol. Consistent with **Figure 2**, the dashed grey vertical line represents 0.25 ng/µl. Egg, untreated egg; EGGb, bleached egg; EGGf, frozen egg; FL, full-length female; LS1, larval stage L1; LS3, larval stage L3; MFL, microfilaria; MIR, miracidia. The number of samples in each comparison is presented in **Table 1**.

We expanded our analysis of the CGP protocol to a total of seven helminth species for which samples were available, including a total of five distinct life stages (**Figure 3**). High variability in mapping was observed between species, with 50% or greater mapping frequency achieved in at least one life stage of five of the seven species tested (**Figure 3A**, **Figure S2A**). Clear differences were observed between multiple life stages tested within a species, likely reflecting differences in extraction efficiency per life stage, for example: for *A. canium*, reads from eggs (median = 54.98) mapped much more effectively than L1 (median = 3.61) or L3 stages (median = 7.57), and in *S. stercoralis*, full-length females (median = 67.84) and L1 (median = 47.19) performed better than L3 (median = 9.01). The proportion of contaminating reads tended to increase as library concentration decreased for all species (**Figure 3B**); however, some samples for which mapping was poor but higher libraries concentrations were obtained were highly enriched for contaminants, for example, *A. canium* L1 larvae and *T. muris* eggs. Interestingly, *T. muris* L1 larvae (median = 41.85) and bleached eggs (median = 51.35) performed much better than untreated eggs (median = 2.72); bleaching is experimentally used for promoting hatching of *T. muris*, by dissolving the egg shell layers and in turn improving access to DNA within. However, bleached eggs were embryonated and developmentally more advanced than untreated unembryonated eggs, and therefore would have more DNA available for library preparation. Similar to untreated *T. muris* eggs, *A. dissimilis* eggs performed poorly (median = 0.16); for both species, few if any nematode sequences were recovered and the majority of sequencing reads were contaminating bacterial-derived contaminants, perhaps indicative of the challenge of accessing material with the environmentally resistant egg. Analysis of *A. dissimilis* was further limited by the lack of a reference genome for this species; we mapped against the available genome of *Ascaris lumbricoides*, the nearest species for which a reference was available, and therefore at best, would have expected suboptimal mapping due to sequence divergence. The duplication rates remain low for all species and conditions tested (**Figure S2B**), consistent with our initial observation that library input did not significantly influence duplication rates (**Figure S1B**).

We also generated data from microfilaria of *D. medinensis*; these samples were extracted using NXT rather than CGP as part of a separate study; however, we have included these for comparison (**Figure S3**). High mapping rates were observed for almost all libraries generated; however, the duplication rates per library were also high (median = 36.90); this was unexpected, given NXT did not produce high duplication rates under the initial conditions tested with *H. contortus* or *S. mansoni*, nor were there excessive PCR cycles used in this instance. Duplication rates were not correlated with library concentration.

In summary, we present successful DNA extraction followed by whole-genome sequencing of individual egg and larval stages from six of eight parasites species examined. These results significantly extend the possibility of genomic analyses for life stages for which, at best, were limited to low-resolution, low-throughput PCR based assays without the addition of whole-genome amplification. Whatman^®^ FTA^®^ cards provide a convenient substrate for sample collection and storage, and do not limit the application of direct DNA extraction and whole-genome sequencing of parasite samples, even for field samples as demonstrated for *S. mansoni* miracidia that were collected and processed in Uganda before they were transported to the UK. Further optimization is required to improve the DNA recovery from eggs, for example, from *A. dissimilis* and *T. muris*, to provide greater applicability of our approaches to species that generate particularly environmentally resistant stages, such as the soil transmitted helminths. The application of whole-genome sequencing to diagnose and monitor helminth infections *at scale* is largely limited by the costs of library preparation and sequencing, and therefore, will be restricted to niche applications of the technology. However, the use of low-coverage whole-genome sequencing with the specific aim to target the mitochondrial genome, which is present at a higher copy number than the nuclear genome (**Table 2**), may be a cost-effective alternative and potentially provides greater diagnostic information than low-throughput PCR-based diagnostics. Continued development of genomic technologies and the associated reduction in sequencing and library preparation costs will make screening large samples by genome sequencing more routine as in viral (Dudas et al., 2017) and bacterial (Domman et al., 2018) population studies. In doing so, the ability to derive high resolution data may provide insight into, for example, (i) within host reproductive dynamics, including within host population size, differential fecundity (Hildebrandt et al., 2014), and reproductive traits such as polyandry (Doyle et al., 2018), (ii) defining parasite transmission zones and rates of transmission between zones to prioritize treatment foci (Crawford et al., 2019; this special edition, in review), (iii) defining effective population sizes of parasite populations (Sallé et al., 2019), and using this to estimate the impact of control strategies over time, and/or (iv) discrimination between reintroduction and recrudescence of parasites in regions where parasite control has been successful (Koala et al., 2019). The use of genomics to provide information-rich data will be increasingly important for diagnostic and surveillance purposes broadly (Cotton et al., 2018), and will be particularly informative as efforts to control human infective helminths using MDA move from control to elimination.

## Data Availability

The datasets generated for this study can be found in ENA, ERP114942.

## Ethics Statement

Given a number of host/parasite systems are described, we have provided information about the individual ethical approvals obtained associated with each species in the “Sample Collection” subheading of the *Methods* section.

## Author Contributions

Conceptualization: SRD, JAC, NH; Methodology: SRD, GS, NH; Software: SRD; Formal analysis: SRD; Investigation: SRD, GS; Resources: FA, TJ, JL, JBC, PC, JW, TC, ET, MD-C, PE, RL, KM, CM, TM, BS, PO, JT, AW, ED, RK, DB, MB; Data curation: SRD, NH; Writing – original draft preparation: SRD; Writing – review and editing: All authors; Visualization: SRD; Supervision: SRD, JAC, NH; Project administration: NH.

## Funding

Work performed at the Wellcome Sanger Institute is supported by the Wellcome Trust (grant 206194) and by the Biotechnology and Biological Sciences Research Council (BB/M003949/1). We thank Alison Elliot for access to *S. mansoni* samples collected from Uganda, the collection of which was supported by the Wellcome Trust (grant 095778/Z/11/Z). *S. mansoni* samples were also obtained from the Schistosomiasis Collection at the Natural History Museum (NHM) [SCAN], which is funded with support from the Wellcome Trust (grant no. 104958/Z/14/Z).

## Conflict of Interest Statement

The authors declare that the research was conducted in the absence of any commercial or financial relationships that could be construed as a potential conflict of interest.

## Supplementary Material

Table S1 Complete metadata per sample (excel workbook)

Figure S1 Summary boxplots of mapped on target reads (A) and duplicate reads (B) for all extraction kits tested. The number of samples tested per kit, per life stage is shown at the top of (A).

Figure S2 Summary boxplots for mapped on target reads (A) and duplicate reads (B) for all species tested using the CGP protocol. The number of samples tested per species, per life stage is shown at the top of (A).

Figure S3 Analysis of *D. medinensis* using the NXT protocol. (A) Mapped “on-target” reads. (B) Duplicate reads. (C) Comparison of the effect of sequencing library concentration on mapping efficiency, colored by the proportion of duplicate reads. (D) Comparison of the effect of sequencing library concentration on mapping efficiency, colored by the proportion of putative contaminant reads identified using Kraken (100 - percent_unclassified_reads).
