# Supplementary figures and images for: Evaluation of DNA Extraction Methods on Individual Helminth Egg and Larval Stages for Whole-Genome Sequencing
Source: Front Genet. 2019 Sep 20;10:826. doi: 10.3389/fgene.2019.00826 (PMC6764475; doi:10.3389/fgene.2019.00826)

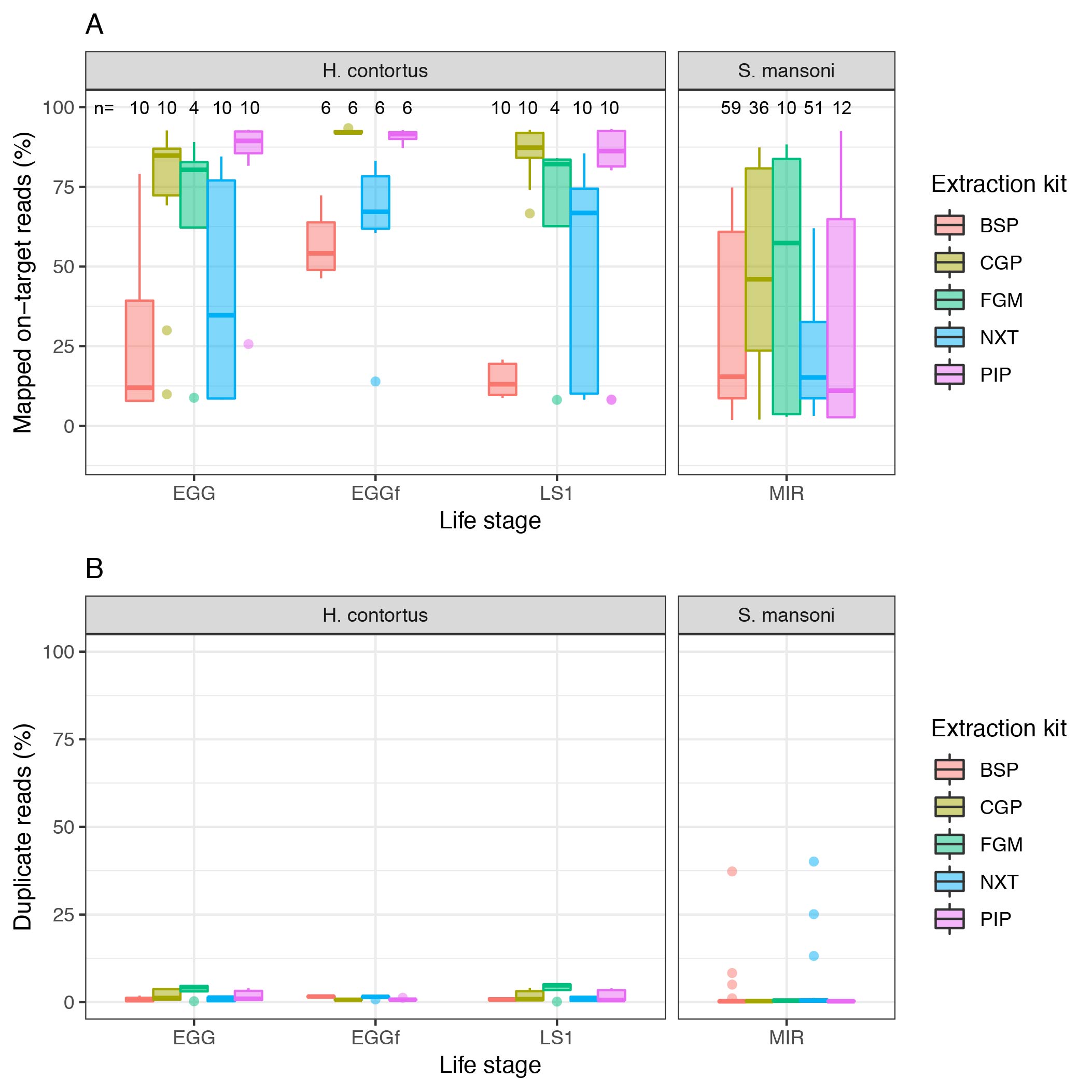

Supplement: Figure S1 — Summary boxplots of mapped on target reads (A) and duplicate reads (B) for all extraction kits tested. The number of samples tested per kit, per life stage is shown at the top of (A). [file Image_1.jpeg]

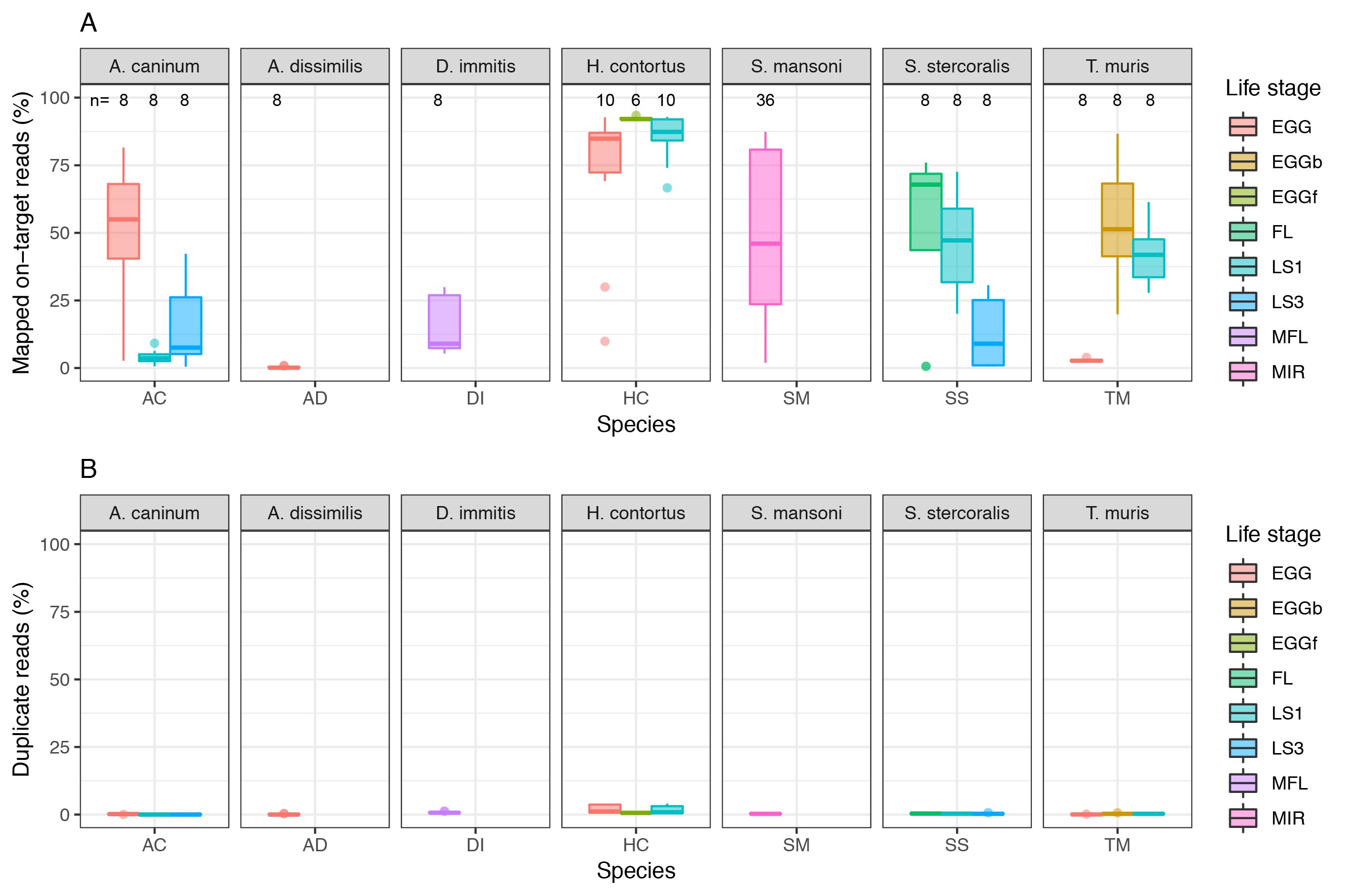

Supplement: Figure S2 — Summary boxplots for mapped on target reads (A) and duplicate reads (B) for all species tested using the CGP protocol. The number of samples tested per species, per life stage is shown at the top of (A). [file Image_2.jpeg]

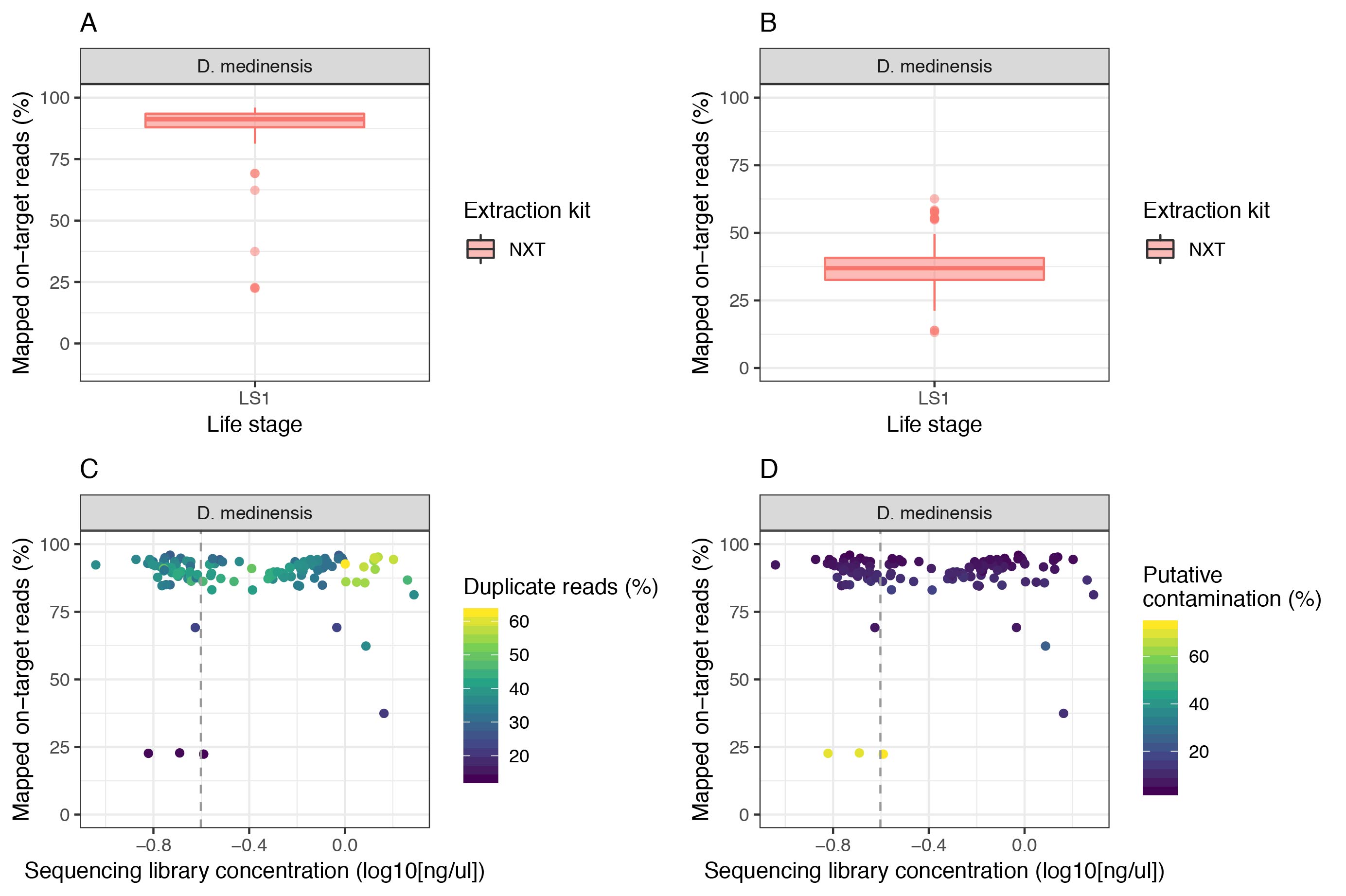

Supplement: Figure S3 — Analysis of D. medinensis using the NXT protocol. (A) Mapped “on-target” reads. (B) Duplicate reads. (C) Comparison of the effect of sequencing library concentration on mapping efficiency, colored by the proportion of duplicate reads. (D) Comparison of the effect of sequencing library concentration on mapping efficiency, colored by the proportion of putative contaminant reads identified using Kraken (100 - percent_unclassified_reads). [file Image_3.jpeg]
